# Differences between human male and female neutrophils in mRNA, translation efficiency, protein, and phosphoprotein profiles

**DOI:** 10.21203/rs.3.rs-4284171/v1

**Published:** 2024-04-23

**Authors:** Darrell Pilling, Kristen M. Consalvo, Sara A. Kirolos, Richard H. Gomer

**Affiliations:** Department of Biology, Texas A&M University, College Station, TX 77843-3474 USA

**Keywords:** Neutrophil, RNA, ribosome, monosome, polysome, sex-based, proteomics, phosphoproteomics, SLIGKV, chemorepulsion

## Abstract

**Background:**

Human males and females show differences in the incidence of neutrophil-associated diseases such as systemic lupus erythematosus, rheumatoid arthritis, and reactive arthritis, and differences in neutrophil physiological responses such as a faster response to the chemorepellent SLIGKV. Little is known about the basis of sex-based differences in human neutrophils.

**Methods:**

Starting with human neutrophils from healthy donors, we used RNA-seq to examine total mRNA profiles, mRNAs not associated with ribosomes and thus not being translated, mRNAs in monosomes, and mRNAs in polysomes and thus heavily translated. We used mass spectrometry systems to identify proteins and phosphoproteins.

**Results:**

There were sex-based differences in the translation of 24 mRNAs. There were 132 proteins with higher levels in male neutrophils; these tended to be associated with RNA regulation, ribosome, and phosphoinositide signaling pathways, whereas 30 proteins with higher levels in female neutrophils were associated with metabolic processes, proteosomes, and phosphatase regulatory proteins. Male neutrophils had increased phosphorylation of 32 proteins. After exposure to SLIGKV, male neutrophils showed a faster response in terms of protein phosphorylation compared to female neutrophils.

**Conclusions:**

Male neutrophils have higher levels of proteins and higher phosphorylation of proteins associated with RNA processing and signaling pathways, while female neutrophils have higher levels of proteins associated with metabolism and proteolytic pathways. This suggests that male neutrophils might be more ready to adapt to a new environment, and female neutrophils might be more effective at responding to pathogens. This may contribute to the observed sex-based differences in neutrophil behavior and neutrophil-associated disease incidence and severity.

## Plain English Summary

Some diseases are more common in females, and this sex difference may be due, in part, to sex differences in immune cells called neutrophils. However, little is known about the basis of sex-based differences in human neutrophils. To understand these differences, we isolated messenger RNA (mRNA) and protein from neutrophils of healthy male and female humans. mRNAs can be efficiently translated, with many ribosomes present on the mRNA translating the mRNA into proteins, or poorly translated, with only one or no ribosomes translating the mRNA into a protein, and for some mRNAs, the translation efficiency is different between male and female neutrophils. We also find that the abundances of many proteins and protein modification by the addition of a phosphate group (phosphorylation), are different between male and female neutrophils. The differences in protein levels and protein phosphorylation suggest that male neutrophils might be more ready to adapt to a new environment, and female neutrophils might be more effective at responding to pathogens. Male neutrophils have more phosphorylated proteins at 5 and 20 minutes after exposure to a compound that regulates neutrophil movement. These differences may contribute to the observed sex-based differences in neutrophil behavior and neutrophil-associated disease incidence and severity.

## Background

Polymorphonuclear cells (neutrophils) are the most abundant circulating immune cell in humans, representing 50–70% of all leukocytes [1, 2], are an important component of the innate immune system [3], and are part of the first line of defense against microorganisms [4]. Neutrophils also have a role in tissue homeostasis, but aberrant activation and persistence can contribute to inflammation and the progression of some disease conditions [3], including acute respiratory distress syndrome (ARDS) [5], rheumatoid arthritis (RA) [6], and many other disorders [7–10].

Sexual dimorphism in the mammalian immune system has been noted for decades [11, 12]. In general, women tend to have stronger innate and adaptive immune responses than men [13–17], including reduced rates of infection and an increased immune response to a variety of bacterial, viral, and parasitic infections [18–21] and some vaccines [22, 23]. However, women also have a higher incidence of autoimmune disorders compared to men [24, 25]. Some of these sex differences can be explained by hormonal differences [26, 27] or sex chromosome copy number [28], but there is much that is still unknown [29].

Circulating neutrophils are heterogenous [30], in part due to significant phenotypic changes during neutrophil maturation and ‘aging’, as well as in response to stimuli/activation [31]. Neutrophil DNA methylation and gene expression show significant inter-individual variations among healthy donors [32]. This inter-individual variation, combined with variable X chromosome inactivation and X inactivation ‘escapism’ (genes on the silenced X chromosome in women that are transcribed) [33], and the influence of sex hormones [27], create a complex system that tightly regulates immune function.

There are differences in mouse neutrophils as a function of sex and age, including differences in chromosomal accessibility, transcriptomics, metabolomics, and lipidomics, resulting in functional differences between male and female neutrophils [34]. In human circulating neutrophils, there are sex-based differences in phenotype and function, with adult female neutrophils having a more activated/mature phenotype, enhanced type I interferon pathway activity, and proinflammatory responses compared to adult male neutrophils [35]. Neutrophils have a distinct proteomic profile compared to other blood immune cells, and neutrophil RNA and protein levels do not necessarily correlate [36–40].

ARDS involves damage to the lungs triggering an influx of neutrophils into the lungs, and the neutrophils then activating, causing further damage to the lungs, and in a positive feedback loop the additional damage recruits more neutrophils [41]. A potential therapeutic modality for ARDS is to use an inhaled neutrophil chemorepellent to drive neutrophils out of the lungs and/or inhibit the entry of neutrophils into the lungs. We found that the peptide SLIGKV-NH2 (hereafter referred to as SLIGKV), a protease activated receptor 2 (PAR2) agonist, is a repellent for human neutrophils, and in a mouse model of. ARDS, aspiration of SLIGKV inhibits the number of neutrophils in the lungs [42]. Surprisingly, compared to human female neutrophils, male neutrophils showed a faster response to SLIGKV [42, 43], and there were several differnces between male and female neutrophils in the signal transduction pathway mediateing chemorepulsion in response to SLIGKV [43].

In this report, we describe, for human neutrophils, sex-based differences in gene expression, translation efficiency, protein abundance, and protein phosphorylation. In response to SLIGKV, we find that at 5 minutes there was increased phosphorylation of two proteins in male neutrophils, but no significantly increased phosphorylation of proteins in female neutrophils. These differences may contribute to the observed sex-based differences in the faster response time of male neutrophils to SLIGKV, and neutrophil-associated disease incidence and severity.

## Methods

### Neutrophil isolation and culture

Human venous blood was collected with the approval from the Texas A&M University Institutional Review Board from healthy volunteers who gave written consent. Neutrophils were isolated at room temperature, as previously described [43]. Cells were resuspended in RPMI-1640 (Lonza, Walkersville, MD) with 2% BSA (Rockland Inc, Limerick, PA) (RPMI-BSA). Cell spots, staining with Giemsa, and quantitation of the percent of neutrophils in the cell preparation were done following [44]. We never used the same donor twice for a given experiment. The age ranges for the donors were 18–44 years for males and 18–32 years for females. Cell preparations were 97.2 ± 0.3% neutrophils. The main contamination cell type was monocytes at 1.1 ± 0.2%, with basophils, eosinophils, and lymphocytes all < 0.6% (**Additional file 1: Fig. S1**). These preparations are of higher purity than preparations previously published for gene expression analysis of neutrophils [34, 35].

### RNA and ribosome collection, fractionation, purification, and sequencing

From each donor, 45 to 115 × 10^6^ unstimulated neutrophils were isolated from whole blood. Samples were treated as described previously [45] with the following modifications. Neutrophils were collected by centrifugation at 500 × g for 5 minutes. Pellets were disrupted by pipetting vigorously with 500 μl ice cold “Complete Polysome Buffer” (15 mM Tris-HCl pH 7.5, 300 mM NaCl, 15 mM MgCl_2_, 1% Triton X-100 (Alfa Aesar, Ward Hill, MA), 100 μg/ml Cycloheximide (VWR, Radnor, PA), 1 mg/ml Heparin (A16198.06, Thermo Scientific, Rockford, IL), 500 units/ml RNasin Ribonuclease inhibitor (Invitrogen, Carlsbad, CA), 20 mM DTT, and 10x Protease and Phosphatase inhibitor cocktail (Thermo Scientific)). Lysed samples were separated on a 10–50% sucrose gradient made with “Polysome Gradient Buffer” (10 mM HEPES-KOH pH 7.5, 70 mM ammonium acetate, 5mM magnesium acetate, and 10 or 50% sucrose) prepared the same day. Cell lysates were layered on top of the prepared sucrose gradient, centrifuged, and then fractionated following the manufacturer’s instructions for a TriAX flow cell (BioComp, Fredericton, New Brunswick, Canada) and FC203B fraction collector (Gilson, Middleton, WI). RNA purification and precipitation was performed as described [45]. Briefly, 0.5 ml of each sucrose fraction was mixed with 0.5 ml TRIzol (Invitrogen) and 0.2 ml chloroform, then clarified by centrifugation at 12,000 × g for 15 minutes at 4°C. 0.5 ml of the upper layer was transferred to a fresh tube containing 1 ml isopropanol and 2 μl of 15 mg/ml Glycoblue (Invitrogen). After mixing, the RNA was precipitated by incubating overnight at −20°C and collected by centrifugation at 12,000 × g for 15 minutes at 4°C. The pellet was rinsed with 1 ml ice-cold 70% ethanol. The ethanol was removed after centrifugation at 12,000 × g for 15 minutes at 4°C. Precipitated samples were re-spun a second time to remove the remaining ethanol from the side of the sample tubes. RNA pellets were air dried for at least 10 minutes at room temperature before being dissolved in 20 μl nuclease-free water (Thermo Scientific). RNA concentrations were checked with a Synergy Mx plate reader with a microdrop attachment (BioTek, Winooski, VT).

RNAseq libraries were created following the manufacturer’s instructions for QuantSeq 3’ mRNA-Seq Library Prep Kit FWD for Illumina (type 015.96, Lexogen Inc, Greenland, NH), with 2 μg of RNA used as the starting material. Libraries were sequenced using an Illumina NextSeq 500 platform (Texas A&M University Institute for Genome Sciences and Society Experimental Genomics Core, College Station, TX). RNA sequencing data were analyzed using the QuantSeq Data Analysis Pipeline on the BlueBee Genomic Platform (BlueBee, San Mateo, CA). Briefly, the quality of sequences was evaluated using FastQC software (version 0.11.5) after adapter trimming with BBDUK software (version 35.92). Gene and transcript intensities were computed using STAR software (version 2.5.2a) with the Gencode Release 27 (GRCh38) human genome as a reference.

For each donor, for each mRNA X, the normalized count of X in the free fraction was calculated as (read count of X in the free fraction)/(total number of read counts in the free fraction).

The normalized count of X in the monosome fractions was similarly calculated as (read count of X in the monosome fraction)/(total number of read counts in the monosome fraction).

Similar normalization was done for early polysomes and late polysomes. The amount of mRNA X in the free mRNA compared to the total amount of mRNA X was then calculated as (normalized read count in the free fraction for mRNA X) / (sum of the normalized read counts for mRNA X in the free, monosome, early polysome, and late polysome fractions).

### Quantitative PCR

RNA reverse transcription and cDNA synthesis were performed as described [45]. Quantitative real-time PCR (qPCR) was performed in a QuantStudio 6 Flex Real-Time PCR System (Life Technologies, Carlsbad, CA). 10 μl reactions were prepared in 96-well plates (MLL9601, BioRad Laboratories, Inc., Hercules, CA) with an AzuraView GreenFast qPCR Blue Mix LR (AZ-2305, Azura Genomics, Raynham, MA) following the manufacturer’s protocols. The relative quantity of *PDE6A* mRNA was calculated using the ΔCT method [46]. GAPDH mRNA was used as a reference [47]. The PCR was performed using 40 cycles and started with 2.5 minutes hold at 95°C followed by 40 cycles of 5 seconds at 95°C, 20 seconds at 60°C, and 15 seconds at 95°C. Primer pairs were, listed 5’ to 3’, modified from previously published work for *GAPDH* [48], or purchased commercially for *PDE6A* (#HP200420; OriGene, Rockville, MD):

*GAPDH* primers: GCACCGTCAAGGCTGAG

CCACTTGATTTTGGAGGGATCTC

*PDE6A* primers: GTCCGTGCTTTCCTCAACTGTG

GGACCAGAGTAAGGTGGAACTTC

### Proteomics, phosphoproteomics, and gene ontology

Proteomics was performed as described [43]. Briefly, in-gel protein preparation of tryptic peptides was performed at the University of Texas Southwestern Proteomics Core (https://proteomics.swmed.edu/wordpress/?page_id=553) for Thermo Fusion Lumos standard gradient mass spectrometry. The proteins were analyzed using Proteome Discoverer 3.0 (Thermo Scientific) and searched using the human protein database from UniProt (www.uniprot.org) [49]. Raw and processed proteomic data was uploaded to MassIVE at the University of California at San Diego Center for Computational Mass Spectrometry (https://massive.ucsd.edu/ProteoSAFe/dataset.jsp?task=002e367a56ef471da06a302861229930) with accession number MSV000088857. For each donor, peptide counts were summed and then divided by the total counts for all peptides from that donor. Male and female values were compared to determine sex-based differential protein abundance.

Isolated neutrophils for phosphoproteomics analysis were prepared as described above. For each condition, 5 × 10^6^ cells were resuspended in 1 mL RPMI-BSA prewarmed to 37°C and then incubated in the presence or absence of 500 ng/ml SLIGKV-NH2 (#3010, Tocris-BioTechne, Minneapolis, MN; SLIGKV) at 37°C in a CO_2_ incubator as described previously [43]. After 5 minutes in the presence or absence of SLIGKV, and 20 minutes in the presence of SLIGKV, cells were placed on ice, tubes were filled with ice cold PBS, and then cells were collected by centrifugation at 500 × g for 5 minutes at 4°C. Cells were then resuspended in ice-cold PBS and recentrifuged. Cell pellets were then resuspended in 0.5 mL RIPA buffer (89900, Thermo Scientific, Waltham, MA) containing 1x protease and phosphatase inhibitors (78441, Thermo Scientific) and incubated on ice for 10 minutes. Lysates were then clarified by centrifugation at 10,000 × g for 10 minutes at 4°C. Supernatants (soluble lysates) and pellets were separated and snap frozen in liquid nitrogen and stored at −80°C. Soluble lysate samples were digested with trypsin and the peptides were analyzed at the UTSW Proteomics Core using Tandem Mass Tag (TMT) quantitation with LC-MS/MS Orbitrap Eclipse mass spectrometry. An aliquot of each sample was run on the Orbitrap Eclipse for the total protein analysis (TMT system). The remaining material was processed using a two-step phosphopeptide enrichment protocol. Samples were first enriched using a High-Select TiO2 Phosphopeptide Enrichment kit (Thermo), and then the flowthrough was collected for secondary enrichment with High-Select Fe-NTA phosphopeptide enrichment columns (Thermo). Each of these steps enriches a different subset of phosphopeptides (with some overlap) leading to a more comprehensive coverage relative to using a single method. The phosphopeptides collected from each enrichment step were then combined and analyzed on the Orbitrap Fusion Lumos. The data were analyzed using Proteome Discoverer 3.0 (Thermo Scientific) using the human protein database from UniProt (www.uniprot.org). Raw and processed proteomic and phosphoproteomics data from the Orbitrap Eclipse mass spectrometry dataset was uploaded to the MassIVE website at the UCSD Center for Computational Mass Spectrometry with accession number MSV000094295.

Differences in protein and phosphopeptide expression between males and females, and between unstimulated and SLIGKV stimulated cells were assessed using t-tests. Fold change in expression and t test values were ranked for volcano plot visualization. Gene ontology (GO) and KEGG (Kyoto Encyclopedia of Genes and Genomes) pathway analysis was performed, and graphs were generated, using ShinyGO (v 0.8 using Ensembl Release 107) [50], and results were confirmed using g:Profiler (https://biit.cs.ut.ee/gprofiler/gost) and Metascape (https://metascape.org/). Groups were analyzed compared with the standard “all proteins” in the Homo sapiens database, and significance (p < 0.05) was determined by Fisher’s exact test with FDR correction. Terms were identified by comparing the list of differentially abundant proteins against the background list of all identified proteins in the proteomics results. Venn diagrams were generated using BioTools (https://www.biotools.fr/misc/venny).

### Whole cell lysate preparation and western blots

Neutrophil whole cell lysates were collected and washed as previously described [43, 44] with the following modifications. A total of 2 × 10^6^ neutrophils in 0.2 ml of RPMI-BSA were washed twice by adding 0.5 ml of room temperature (RT) 1x PBS before the cells were collected by centrifugation at 500 × g for 5 minutes at RT, and the supernatant was removed. The cells were then resuspended in 0.1 ml of 1x SDS sample buffer with 2-ME with 10x protease and phosphatase inhibitor cocktail (1861281; Thermo Scientific) and pipetted vigorously to resuspend and lyse the cells, and heated for 5 minutes at 98°C. Western blots were stained with 2.3 μg/ml anti-CYFIP1 (NBP2-92695; Novus Biologicals, Littleton, CO), 0.05 μg/ml anti-NAP1 (NBP2-24727SS; Novus), or 0.1 μg/ml anti-GAPDH mouse mAb (60004-1-Ig; Proteintech, Rosemont, IL) following the manufacturer’s protocols. Bound antibodies were detected with an ECL Western blotting kit (Thermo Scientific). On each experiment day, neutrophils from one male and one female were collected. Western blot band intensities were quantified using Image Lab software (Bio-Rad Laboratories, Hercules, CA) and normalized to each test sample’s GAPDH loading control, and the ratio for the female donor was normalizing to the ratio for the date-matched male donor.

### Fixed-cell microscopy

Fixed-cell microscopy of unstimulated neutrophils was performed as previously described [43] with the exception that cells were incubated overnight at 4°C in a humid chamber with 4.7 μg/ml anti-CYFIP1 or 0.05 μg/ml anti-NAP1 in PBS/0.1% Tween 20. Immunofluorescence images were captured with a 40x objective using a Ti-Eclipse inverted fluorescence microscope (Nikon, Tokyo, Japan). Mean fluorescence intensity (MFI) of all neutrophils in a field of view (>10 cells per field of view with an average of five or more fields of view per antibody per donor) was quantified as described [43].

### Statistics

Prism v7 (GraphPad Software Inc., San Diego, CA, USA) and Microsoft 365 Excel (Microsoft, Redmond, WA) were used for data analysis. Graphs were generated with Prism. Data are shown as mean ± SEM except where otherwise stated. To determine whether the mean difference between two groups was statistically significant, the Mann-Whitney test was used. Statistical significance was defined as p 0.05. For the volcano plots, one unpaired t test per row was calculated, without assuming consistent SD (the fewer assumptions option), with an uncorrected significance of p < 0.05. GO term groups were analyzed compared with the standard “all proteins” in the Homo sapiens database, and significance (p < 0.05) was determined by Fisher’s exact test with FDR correction.

## Results

### Male and female neutrophils show differences in translation efficiency of some mRNAs

Changes in the levels of many mRNAs have a poor correlation with changes in the levels of the proteins they encode, indicating that for some proteins, levels are regulated by changes in protein stability or changes in the extent to which their encoding mRNAs are translated [51]. The latter can be assessed by ribosome fractionation analysis or ribosome profiling [52], where poorly translated mRNAs are not bound to ribosomes (free mRNA), or bind a single ribosome (monosome), while strongly translated mRNAs are found associated with multiple ribosomes (polysomes). Polysome fractionation and profiling has been used to analyze translation efficiency in human monocyte-derive macrophages [53], neutrophil-like differentiated HL-60 myelocytic cells [54], platelets [55], a mouse promyelocyte cell line [56] and macrophages [57]. To assess translation efficiency in circulating human neutrophils, we isolated neutrophils from 3 male and 4 female healthy donors, lysed the cells, and separated the lysates on sucrose gradients as described in [45] and determined profile features as described in [52]. Fractionated male and female neutrophils, despite showing donor to donor variations in the profiles, all contained a clear monosome peak (**Additional file 2: Fig. S2**). Similar experiments on the human MCF7 cancer cell line also showed replicate experimental variation in the ribosome profiles [58]. The coefficient of variation (Standard Deviation / Mean) for the polysome region (defined as gradient position 40 – 75, consisting of fractions 7 – 12), showed no significant difference between the male and female profiles. These profiles show some indication of peaks in the polysome regions for both male and female neutrophils, most clearly seen in male donor #2 and female donors #1 and #4 (**Additional file 2: Fig. S2**). Neutrophils have a significantly lower resting gene expression profile than other immune cell types, such as peripheral blood mononuclear cells [59], with low but detectable transcriptional activity [60, 61], which increases rapidly after neutrophil activation [60]. This reduced basal transcription activity may be responsible for the low polysome peaks.

**Figure 1:**
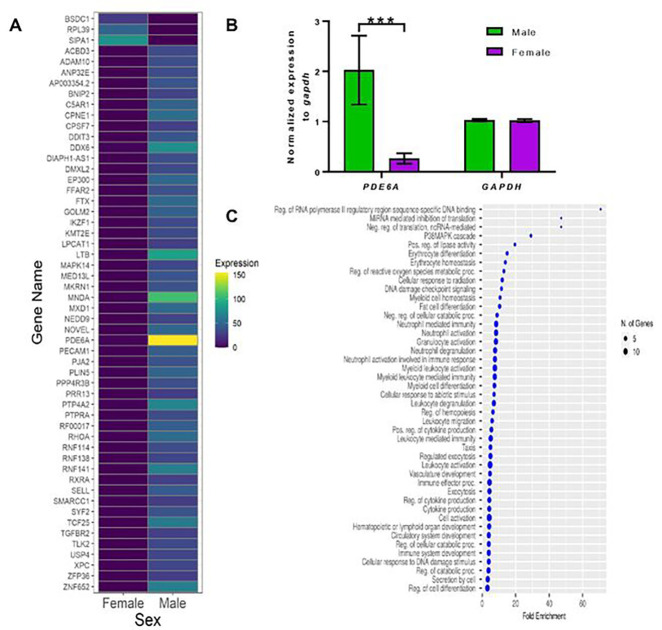
Male and female neutrophils have differences in the levels of some mRNAs. **A)** RNA-seq of total mRNA identified mRNAs that are enriched in male or female neutrophils. **B)**RNAs from neutrophil whole cell lysates were analyzed for relative gene expression of *PDE6A* and *GAPDH* using qPCR. Gene expression levels are normalized to *gapdh* as a control. Values are from 2 male donors and 4 female donors. *** indicates p 0.001 (2-way ANOVA, Tukey’s test. **C)** Gene ontology analysis indicates that some mRNAs present at higher levels in male neutrophils encode proteins associated with translation regulation and immune cell activation/degranulation.

There are sex-based transcriptomic differences, based on analysis of RNA-seq of total mRNA, in human [35, 62] and murine bone marrow-derived neutrophils [34]. In human neutrophils, 106 genes were upregulated and 128 genes were downregulated in female compared to male neutrophils [35]. In agreement with that work, we observed, using RNA-seq of total mRNA, sex-based differences in the levels of some mRNAs in human neutrophils from 2 male and 4 female donors(Fig. 1A and **Additional file 3: Table S1; Tab1**). Increased levels of one of the mRNAs, phosphodiesterase 6A (*PDE6A*), observed to be present at higher levels in male neutrophils, was verified by qPCR with *GAPDH* as a control (Fig. 1B).

To assess translation efficiency, male and female neutrophils were fractionated, and RNA-seq was done for free mRNA (Fractions 1 – 3, corresponding to gradient positions 1 – 20 in **Additional file 2: Fig. S2**), monosomes (Fractions 4 – 6, corresponding to gradient positions 21 – 40 in **Additional file 2: Fig. S2**), early polysomes (Fractions 7 – 9, corresponding to gradient positions 41 – 55 in **Additional file 2: Fig. S2**), and late polysomes (Fractions 10 – 12, corresponding to gradient positions 56 – 75 in **Additional file 2: Fig. S2**).

Examining the amount of each mRNA in the free mRNA compared to the total amount of that mRNA, and then comparing this value for males to the value for females, there were 12 mRNAs with greater abundance in the free fraction in males, and seven with greater abundance in females (**Additional file 3: Table S1; Tab 2**). Similar analysis identified 15 mRNAs with greater abundance in the monosome fraction in males, and four with greater abundance in females (**Additional file 3: Table S1; Tab 3**). There were 22 mRNAs with greater abundance in the early polysome fraction in males, and 22 with greater abundance in females (**Additional file 3: Table S1; Tab 4**). There were 44 mRNAs with greater abundance in the late polysome fraction in males, and seven with greater abundance in females (**Additional file 3: Table S1; Tab 5**).

To further elucidate sex-based differences in the translation of neutrophil mRNAs, each mRNA X for each donor was assessed for Translation Rate (TR_X_) using

TR_X_ = (Early Polysome + Late Polysome)/(Free RNA + Monosome)

Further analysis was then done for mRNAs where all 3 male donors had a non-infinite value for TRX, and the mean and standard deviation was calculated for the TR_X_ value for each mRNA. Only those mRNAs with (standard deviation / mean) < 0.5 were considered for further analysis (**Additional file 4: Table S2**). For males, 163 mRNAs were identified using these criteria, with an average TR_X_ of 2.0 ± 0.4. The highest TR_X_ (and thus the mRNA with the highest percentage of the mRNAs in polysomes) was adenosylhomocysteinase like 1 (AHCYL1, ENSG00000168710) with a TR_X_ of 34.5 ± 6.6 and the lowest TR_X_ (and thus the mRNA with the lowest percentage of the mRNAs in polysomes) was lysine methyltransferase 2B (KMT2B, ENSG00000272333) with a TR_X_ of 0.05 ± 0.01 (**Additional file 4: Table S2; Tab 1**). Of these 163 mRNAs, nine had significantly different TR_X_ values (and thus different percentages of the mRNA in polysomes) between males and females.

A similar analysis was then done for female TR_X_ values. 55 mRNAs were identified with an average TR_X_ of 1.6 ± 0.3. The highest TR_X_ was S100 calcium binding protein A9 (S100A9, ENSG00000163220) with a TR_X_ of 6.7 ± 0.4 and the lowest TR_X_ was signal transducer and activator of transcription 3 (STAT3, ENSG00000168610) with a TR_X_ of 0.06 ± 0.01. Of these 55 mRNAs, only three had statistically different ratios between males and females (**Additional file 4: Table S2; Tab 2**). The three mRNAs were RNA binding motif protein 25 (RBM25, ENSG00000119707), bromodomain adjacent to zinc finger domain 1A (BAZ1A, ENSG00000198604), and bromodomain adjacent to zinc finger domain 2B (BAZ2B, ENSG00000123636). There were 12 mRNAs in both the male and female TR_X_ lists, with BAZ1A and BAZ2B present in both lists (**Additional file 4: Table S2; Tabs 1 and 2**).

To further elucidate sex-based differences in strong translation of neutrophil mRNAs, each mRNA X of each donor was assessed for Strong Translation Rate (STR_X_) using

STR_X_= (Late Polysome)/(Free RNA + Monosome + Early Polysome)

Analysis for STR_X_ was performed similarly to TR_X_, described above (**Additional file 4: Table S2; Tabs 3 and 4**). For the male strongly translated mRNAs, 129 mRNAs were identified with an average ratio mean of 0.64 ± 0.14. The highest ratio was mitochondrially encoded cytochrome C oxidase III (MT-CO3, ENSG00000198938) with a mean STR_X_ of 8.8 ± 2.2 and the lowest qualifying ratio was bromodomain adjacent to zinc finger domain 2B (BAZ2B, ENSG00000123636) with a mean STR_X_ of 0.030 ± 0.004. Of these 129 mRNAs, 13 had significantly different STRX ratios between males and females (**Additional file 4: Table S2; Tab 3**).

Finally, a similar analysis was then done for female STR_X_ values. 46 mRNAs were identified with an average STR_X_ of 0.73 ± 0.14. The highest ratio was mitochondrially encoded tRNA-Val (GUN) (MT-TV, ENSG00000210077) with a STR_X_ of 7.2 ± 1.7 and the lowest qualifying ratio was GABA type A receptor-associated protein (GABARAP, ENSG00000170296) with a STR_X_ of 0.03 ± 0.01. Of these 46 mRNAs, five had significantly different STR_X_ ratios between males and females (**Additional file 4: Table S2; Tab 5**). Combining the TR_X_ and the STR_X_ results, there is more translation of at least 16 mRNAs in human male neutrophils, and more translation of at least 8 mRNAs in female neutrophils. Of the 16 mRNAs that had higher translation efficiency in male neutrophils, 8 encode RNA binding proteins (QKI, RPS15, RBM39, RPL27, MKRN1, RPGR, PSIP1, and ANXA2) and of the 8 mRNAs with higher translation efficiency in female neutrophils, 3 encode cytoskeletal binding proteins (HCLS1, MYH9, and VAPA) and one mRNA encodes a ubiquitin hydrolase (USP15).

### Male and female neutrophils show differences in levels of some proteins

**Figure 2:**
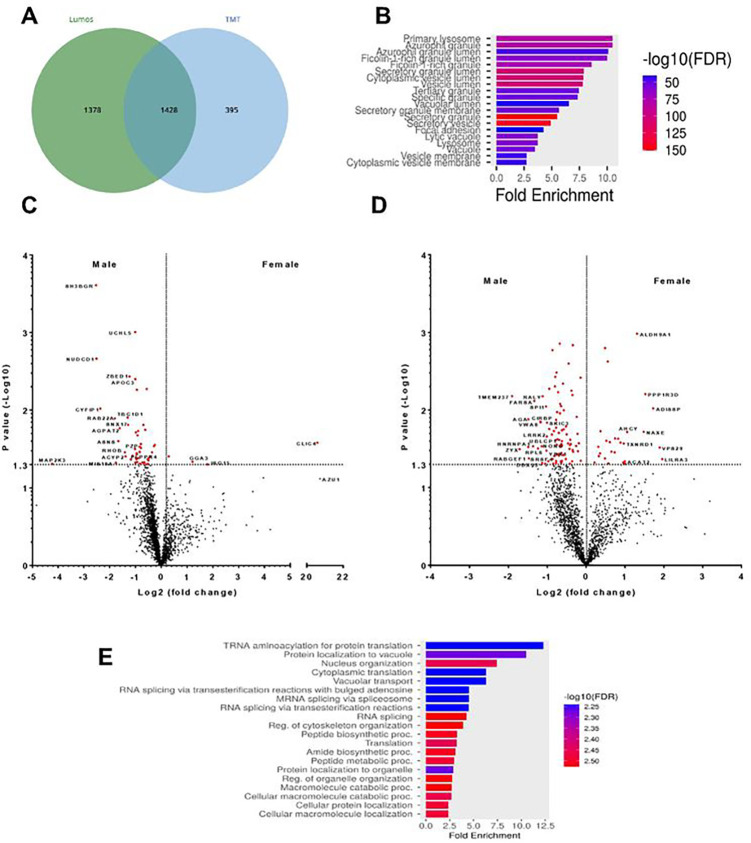
Comparison of proteomics from 2 independent datasets. **A)**Comparison of individual proteins identified by Lumos and TMT-Orbitrap mass spectrometry. **B)** Gene ontology analysis of the 1428 proteins identified in both datasets indicates protein enrichment related to primary and secretory granules, and lysosomal proteins. Volcano plots from **C)** Lumos and **D)** Orbitrap datasets showing the fold change (Log2) and p-value (−Log10) comparing the proteomes from male and female donors. Proteins are marked in red have p values <0.05 (−Log10 >1.3), with those proteins having more than a two-fold change (Log2 <-1 or >1) indicated with gene ID. **E)** GO term KEGG analysis of combined proteins enriched in male proteins.

To determine if the observed sex-based differences in mRNAs and mRNA translation efficiencies are associated with differences in protein abundances, unstimulated neutrophils were analyzed by proteomics using Thermo Fusion Lumos gradient mass spectrometry, and this identified 2806 proteins. We also analyzed neutrophil proteins with TMT LC-MS/MS Orbitrap Eclipse mass spectrometry, and this detected 1,823 individual proteins, with 1,428 proteins identified in both the Lumos and TMT Orbitrap datasets (Fig. 2A).

The most abundant proteins detected in the 1,428 proteins identified in both the Lumos and TMT Orbitrap datasets included myeloperoxidase (MPO), neutrophil elastase (NE), the neutrophil serine protease inhibitor SERPINB1, azurocidin (AZU1), the neutrophil gelatinase-associated lipocalin (LCN2), and S100A8 (**Additional file 5: Fig. S3A)**. These are all proteins that are highly expressed in neutrophils [63, 64], and none of these were higher in males or females. GO term pathway analysis of the 1,428 proteins present in both datasets (Fig. 2A) identified proteins found in neutrophil granules (MPO, LYZ, CTSG, and LTF), and proteins involved with adhesion (RHOA, ACTN1, VIM, and EZR), and lysosomes and vacuoles (RAB2A, VPS18, and LAMP2) (Fig. 2B). Proteins expressed by monocytes such as CD14, CD32a, CD33 and CD58, by lymphocytes such as CD82, by NK cells such as CD16a, by platelets such as CD63 and CD66b, and by B cells and dendritic cells such as CD48/SLAMF2, had either very low levels or were undetectable (**Additional file 5: Fig. S3B)**. Similar analysis of the proteins in just the Lumos or just the Orbitrap datasets also showed enrichment for neutrophil proteins and very little, if any, proteins associated with monocytes, lymphocytes, NK cells, platelets, B cells, or dendritic cells (**Additional file 6: Table S3 Tabs 1–3**). These results are consistent with the cell counts (**Additional file 1: Fig. S1**) indicating that the cell preparations were highly enriched for neutrophils.

In the Lumos dataset, 52 proteins had sex-based differences in protein abundance, with 48 proteins more abundant in male neutrophils and 4 proteins more abundant in female neutrophils (Fig. 2C and **Additional file 6: Table S3 Tab 1**). In the TMT Orbitrap dataset, 112 proteins had sex-based differences in protein abundance, with 85 proteins more abundant in male neutrophils and 27 proteins more abundant in female neutrophils (Fig. 2D, **Additional file 6: Table S3 Tab 2, and Additional file 5: Fig. S3C**). Comparing the two proteomics sets, there was one protein that was higher in females in the Lumos set but lower in females in the TMT Orbitrap set, and this was excluded from further analysis. Proteins that were higher in one sex or the other in the Lumos dataset were either not present, or the data were not significant (generally because the peptide counts were low), in the TMT Orbitrap dataset, and vice versa. Combining the two proteomics datasets, there were 132 proteins more abundant in male neutrophils and 30 proteins more abundant in female neutrophils (**Additional file 6: Table S3 Tab 3**). Surprisingly, none of the 24 proteins encoded by mRNAs where there was a significant sex-based difference in translation efficiency of the mRNA (**Additional file 4: Table S2; Tab 5**) showed a significant sex-based difference in levels of the associated protein.

KEGG and GO term pathway analysis of the 132 male enriched proteins (Fig. 2E and **Additional file 6: Table S3 Tab 3**) identified 23 proteins involved with the spliceosome, nucleocytoplasmic transport, aminoacyl-tRNA biosynthesis, and the ribosome. These include 12 proteins involved with the spliceosome and nucleo-cytoplasmic transport (ALYREF, SNRNP200, LSM4, HNRNPA1, HNRNPC, HNRNPU, HSPA8, MAGOHB, SRSF4, SNRPA, TPR, and NUP93), 5 aminoacyl-tRNA synthetases (EPRS1, FARSA, HARS1, IARS1, and NARS1), and 6 ribosomal proteins (RPL6, RPL15, RPL23A, RPL36A, RPS3, and RPS15A). There were also 6 proteins involved with inositol phosphate metabolism and phosphatidylinositol signaling (INPP1, ALDH6A1, MTMR14, PIP4K2C, PPIP5K2, and DGKZ). The other male enriched proteins were in a variety of additional pathways (Fig. 2E and **Additional file 6: Table S3 Tab 3**).

The 30 female enriched proteins (**Additional file 6: Table S3 Tab 3**) were enriched for proteins involved in a variety of cytosolic metabolic processes (ALDH9A1, ACAT2, AHCY, and EPHX1), endosome/lysosome/proteosome proteolytic pathways (AHCY, EPHX1, TOLLIP, RAD23B, and GGA3), and serine/threonine phosphatase regulatory proteins (PPP1R3D and PPP2R2A).

**Figure 3:**
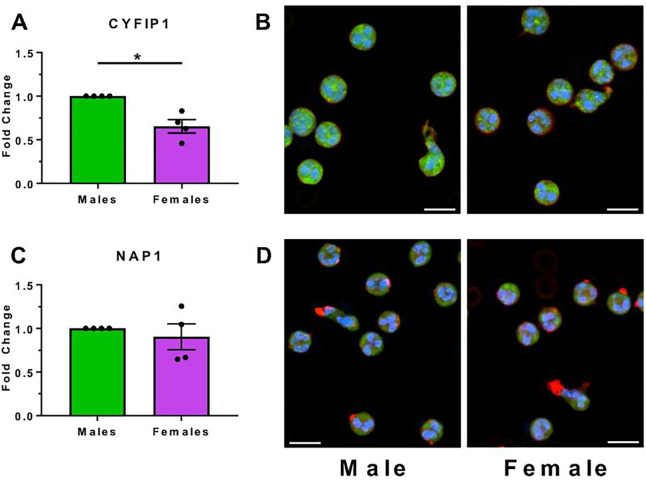
CYFIP1 is enriched in male neutrophils. Unstimulated male and female neutrophil lysates were analyzed by western blots to quantify levels of **A)** CYFIP1, or **D)** NAP1. Bars and error bars are mean ± SEM from four males and four females. Unstimulated neutrophils were fixed, permeabilized, and stained for F-actin (red), and either **B)** CYFIP1, or **E)**NAP1 (green). Blue is DAPI staining of DNA. Bars are 10 μm. **C and F**) Quantitation of the mean fluorescence intensity (green) in **B** and **E)**, respectively, normalized to the average mean fluorescence of each experiment’s male (one male and one female were used for each individual experiment) for each antibody. Images and quantitation are representative of three (NAP1) or four (CYFIP1) independent experiments. * indicates p < 0.05 (Mann-Whitney *U* test).

In the Lumos dataset, cytoplasmic FMR1-interacting protein 1 (CYFIP1; UniProt Q7L576) is one of the 85 proteins that were more abundant in male neutrophils (**Additional file 6: Table S3 Tabs 1 and 3**). In agreement with the proteomics results, CYFIP1 was more abundant in male neutrophils both by Western blots (Fig. 3A) and immunofluorescence staining (Fig. 3B–C). The proteomics indicated no sex-based differences in the abundance of Nck-associated protein 1 (NAP1; UniProt Q9Y2A7; also known as NAP125, NCKAP1, or HEM1) (**Additional file 6: Table S3 Tab 1**), and this was also observed by Western blots and immunofluorescence (Fig. 3D–F).

**Figure 4:**
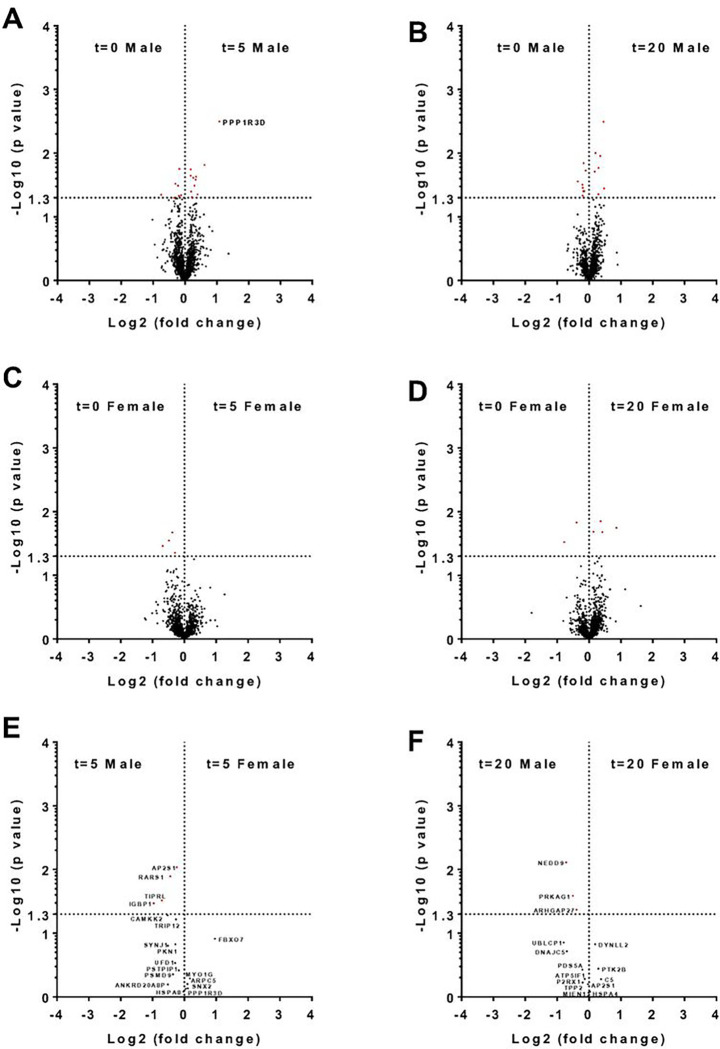
Comparison of proteomics from unstimulated and SLIGKV-stimulated neutrophils. Volcano plots showing the fold change (Log2) and p value (−Log10) comparing significant differences in total protein abundance in **A)** unstimulated versus 5 minutes stimulated male cells, **B)** unstimulated and 5 minute stimulated female cells, **C)** unstimulated and 20 minute stimulated male cells, and **D)**unstimulated and 20 minute stimulated SLIGKV stimulated female cells. Proteins are marked in red have p values <0.05 (−Log10 >1.3), with those proteins having more than a two-fold change (Log2 <-1 or >1) indicated with gene ID. **E)** Volcano plot showing proteins with significant difference in abundance after 5 minutes with SLIGKV in male cells (red dots in **A)** versus females after 5 minutes (red dots in **C**). **F)** Volcano plot showing proteins with significant differences in abundance after 20 minutes with SLIGKV in male cells (red dots in **B**) versus females after 20 minutes (red dots in **D**).

To determine if the more rapid response of male neutrophils to the chemorepellent SLIGKV [43] corresponds to a more rapid change in protein levels, neutrophils were incubated with SLIGKV. After 5 or 20 minutes, only the protein phosphatase PPP1R3D showed a greater than 2-fold change in total protein levels, and this occurred in male neutrophils (Fig. 4A–D). We assessed if proteins that had a difference in protein abundance, irrespective of fold change, in males after 5 minutes incubation with SLIGKV (red dots in Fig. 4A) were also significantly changed in females after 5 minutes (red dots in Fig. 4C). Four proteins (AP2S1, RARS1, TIPRL, and IGBP1) were elevated in male compared to female cells (Fig. 4E). We also determined if the proteins that showed a significant change in levels in male neutrophils after 20 minutes incubation with SLIGKV (red dots in Fig. 4B) were also significantly changed in females after 20 minutes (red dots in Fig. 4D). Three proteins (NEDD9, PRKAG1, and ARHGAP27) were elevated in male compared to female cells (Fig. 4F). Together, the data indicate that SLIGKV affects levels of proteins in both male and female neutrophils within 5 minutes, but a comparison of the number of proteins with significantly changed levels (number of red dots in Fig. 4A, C) suggests that more proteins show changes in levels in male neutrophils. A similar effect was observed at 20 minutes (number of red dots in Fig. 4B and D).

### Male and female neutrophils show differences in protein phosphorylation

To determine if the observed sex-based differences in mRNAs and proteins are also associated with differences in protein phosphorylation, neutrophil proteins were digested with trypsin, the phosphorylated peptides were purified, and these peptides were analyzed to identify phosphorylated proteins. There was no significant difference in the total number of phosphoproteins identified in male and female neutrophils (**Additional file 7: Figs. S4A and B**). A total of 396 phosphoproteins were identified from male and female donors. GO term analysis of these phosphoproteins indicated enrichment for neutrophil and myeloid mediated immunity, including degranulation, activation, and exocytosis (**Fig. S4C**). The phosphoproteins included many common neutrophil proteins, such as MPO, S100A9, LTF, and AZU (**Additional file 6: Table S3 Tab 4**). These phosphoproteins included 22 proteins encoded on the X chromosome including proteins involved in RNA processing (NKAP, HTATSF1, DKC1, RBMX2, MSN, MECP2, FLNA, and TMSB4X), and cellular activation (WAS, DKC1, MSN, MECP2, FLNA, NKAP, ELF4, SASH3, SH3KBP1, and PGRMC1). There were no Y chromosome-encoded phosphoproteins.

**Figure 5:**
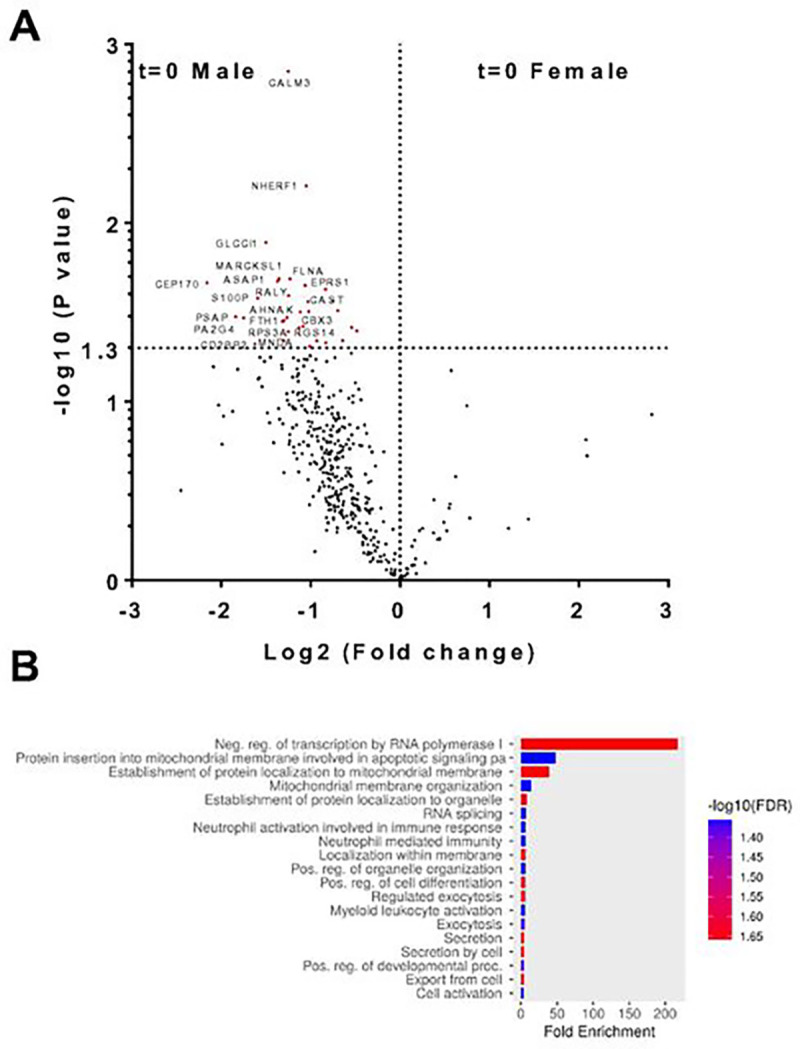
Comparison of phosphoproteomics from unstimulated and SLIGKV-stimulated neutrophils. **A)** Volcano plot comparing phosphoproteins from unstimulated male and female neutrophils. Proteins having p values <0.05 (−Log10 >1.3) and more than a two-fold change (Log2 <-1 or >1) are indicated with gene ID and marked in red. **B)** GO term analysis of phosphoproteins enriched in unstimulated male cells.

Of the 396 phosphoproteins identified in unstimulated neutrophils, 32 of the phosphoproteins had a significant and > 2-fold sex-based difference in phosphorylation, with all 32 phosphoproteins being more phosphorylated in male neutrophils (Fig. 5A and **Additional file 5: Table S3 Tab 5**). The 32 phosphoproteins were enriched for proteins that inhibit transcription by RNA polymerase I and regulate RNA splicing (MACROH2A1, AHNAK, RALY, MFAP1, SRRM2, and CD2BP2), regulate protein localization and apoptotic signaling in mitochondria (BAD, NMT1, RPS3A, CALM3, and FLNA), and regulate neutrophil activation (MNDA, S100P, FTH1, PA2G4, and PSAP) (Fig. 5B, **Additional file 8: Fig. S5A, S5B, and Additional file 5: Table S3 Tab 6**). Of the 32 proteins with a sex-based difference in phosphorylation, 30 showed no significant sex-based difference in total protein abundance. Only 2 proteins with a sex-based difference in phosphorylation (EPRS1 and RALY) had a significant sex-based difference in total protein abundance; both showed increased phosphorylation in males, and increased abundance in males (Additional file 5: TableS3 Tab3).

At 5 minutes, SLIGKV increased phosphorylation of TMC8 and NUP188 in male neutrophils, and SLIGKV did not significantly decrease phosphorylation of any detected proteins in males (**Additional file 9: Fig. S6A, Additional file 8: Fig. S5C-S5D**). There was no significant effect of SLIGKV on protein phosphorylation in female neutrophils at 5 minutes (**Additional file 9: Fig. S6B**). SLIGKV did not significantly affect total protein levels of TMC8 and NUP188 at 5 minutes (Fig. 4C, 4D, **Additional file 6: Table S3 Tabs 1–2**). TMC8 (also called EVER2) is a ion channel-like transmembrane protein associated with the ER and Golgi with higher expression in keratinocytes and immune cells including neutrophils (www.proteinatlas.org), and elevated levels of TMC8 are associated with increased numbers of immune cells in tumors [65]. Mutations in TMC8 are associated with Epidermodysplasia verruciformis [66]. NUP188 is a component of the nuclear pore complex (NPC), regulates chromosome segregation, and NUP188 mutations are associated with a variety of inherited genetic syndromes and cancers [67–70].

At 20 minutes, SLIGKV increased phosphorylation of HNRNPH1 in male neutrophils, did not significantly decrease phosphorylation of any detected proteins in males (**Additional file 9: Fig. S6C, S5E**), and had no significant effect on protein phosphorylation in female neutrophils (**Additional file 9: Fig. S6D**). SLIGKV did not significantly affect total protein levels of HNRNPH1 at 20 minutes (Fig. 4C, 4D, **Additional file 6: Table S3 Tabs 1–2**). NHRNPH1 is an RNA binding protein that associates with pre-mRNAs in the nucleus and regulates mRNA processing and splicing [71]. The only protein showing higher phosphorylation in female neutrophils was PRUNE2, and the phosphorylation was only significantly higher at 20 minutes after SLIGKV exposure (**Additional file 8: Fig. S5F**). There was no significant difference in total protein levels of PRUNE2 (Fig. 4D, **Additional file 6: Table S3 Tab 1**). PRUNE2 (also called BMCC1), suppresses RHOA and AKT signaling, reducing cell migration and survival [72, 73]. It is unclear how phosphorylation of PRUNE2 affects its function. Together these data indicate that SLIGKV affects protein phosphorylation in male but not female neutrophils at 5 and 20 minutes, in agreement with the faster responses of male neutrophils to SLIGKV [43].

## Discussion

Our data indicate that, as previously observed, [34, 35] male and female neutrophils have sex-based differences in levels of some mRNAs. Although there were sex-based differences in the translation efficiency of 24 mRNAs, the encoded proteins did not show sex-based differences in protein levels. One possibility is that for these proteins, a sex-based increased translation rate might be offset by an increased sex-based degradation rate, resulting in similar levels of the proteins in male and female neutrophils.

Human male neutrophils have higher levels of many mRNAs, with GO terms including regulation of RNA metabolic processes and leukocyte chemotaxis [74], while female neutrophils also have higher levels of many mRNAs, with GO terms including type I interferon stimulated genes [35]. We observed 132 proteins that were more abundant in unstimulated male neutrophils and 30 proteins were more abundant in unstimulated female neutrophils. In male neutrophils, many of the 132 upregulated proteins are involved with translation (tRNA biosynthesis, spliceosome regulation, and RNA and ribosome binding), inositol phosphate metabolism, and phosphatidylinositol signaling. CYFIP1 was more abundant in male neutrophils and interacts with translation initiation factor eIF4E [75], suggesting the intriguing possibility that changes in levels of CYFIP1 may account for some of the observed sex-based differences in translation in neutrophils. CYFIP1 also regulates the actin cytoskeleton [76–79], suggesting that changes in levels of CYFIP1 may account for some of the observed sex-based differences in neutrophil chemorepulsion. The 30 proteins with higher levels in female neutrophils were enriched for proteins present in granules, metabolic processes, and proteolytic pathways, but were generally not encoded by type I interferon stimulated genes. This is in agreement with the observation that mRNA and protein levels often do not correlate [39, 80, 81]. These data may help to explain observations that female neutrophils have a higher phagocytic activity and a more effective immune response to infection [14, 82]. In male neutrophils, there was an enrichment of mRNAs and proteins involved with translation, whereas female neutrophils were enriched for mRNAs and proteins involved with metabolic, proteolytic, and cytoskeletal pathways. These data may also help explain the observation that male neutrophils have an “immature” profile, suggesting recent release from the bone marrow and still undergoing differentiation with residual translation, whereas female neutrophils have a more mature profile and are primed for granule release and response to infections [34, 35, 62, 82].

We previously observed that male neutrophils have a more rapid response to the chemorepellent SLIGKV [43]. We found that there were 5 proteins that were elevated in male neutrophils at 5 minutes after incubation with SLIGKV, and no proteins elevated at 5 minutes in female neutrophils. Protein phosphatase 1 regulatory subunit 3D (PPP1R3D) was enriched in unstimulated female neutrophils but showed a significant increase in protein levels in male neutrophils after 5 minutes with SLIGKV. PPP1R3D is a regulatory subunit of protein phosphatase 1 (PP1), which regulates many cellular processes including cell polarization and migration [83, 84]. Four other proteins (AP2S1, TIPRL, IGBP1, and RARS1) were also elevated in male neutrophils at 5 minutes. AP2S1 is a component of the adaptor protein complex 2 (AP-2) which is involved with clathrin-dependent endocytosis [85], TIP41-like protein (TIPRL) in an inhibitor of the protein phosphatases 2A and 4 [86], immunoglobulin-binding protein 1 (IGBP1) binds the protein phosphatase PP2A and protects it from degradation [87], and cytoplasmic Arginine-tRNA ligase (RARS1) is a tRNA synthetase involved in translation [88]. Besides translation, RARS1 is also involved in the arginylation of β-actin by arginyl-tRNA protein transferase 1 (ATE1) at the leading edge of migrating cells [89, 90]. Together, this suggests that the fast response to SLIGKV in male neutrophils may be due to effects on protein phosphorylation, endocytosis, and motility. The fast increase in levels of these proteins is difficult to explain by an increase in protein synthesis, so one possibility is that SLIGKV induces a very rapid inhibition of the degradation of these proteins in male but not female neutrophils.

After 20 minutes incubation with SLIGKV, three proteins (NEDD9, PRKAG1, and ARHGAP27) were elevated in male compared to female neutrophils, and no proteins were significantly elevated in female neutrophils. Enhancer of filamentation 1 (hEF1, NEDD9) is an adaptor protein involved in adhesion and cell migration [91], 5’-AMP-activated protein kinase subunit gamma-1 (PRKAG1) is a regulatory subunit of the AMP-activated protein kinase (AMPK), which not only regulates biosynthesis of fatty acid and cholesterol but also cell migration [92], and Rho GTPase-activating protein 27 (ARHGAP27) is a member of the Rho/Rac/Cdc42-like GTPase activating (RhoGAP) protein family, which regulates cell motility [93]. ARHGAP27 is in a locus for susceptibility to SLE, which is more prevalent in females [94, 95]. These data suggest that although at 20 minutes, both male and female neutrophils move away from the chemorepellent SLIGKV[43], male neutrophils also upregulate proteins involved with cell motility.

The 32 proteins showing increased phosphorylation in male neutrophils include proteins that regulate processing of RNA (AHNAK, HNRNPH1, and RALY), proteins that transport molecules between the cytoplasm and nucleus (NUP188), and proteins such as calmodulins and actin binding proteins that regulate signaling and cell migration (CALM3, TMC8, and FLNA). Filamin-A (FLNA) is an X-chromosome encoded actin-binding protein that cross links actin and links membrane proteins to the cytoskeleton [96]. Phosphorylation of FLNA positively regulates cell migration in many cells, including neutrophils [97, 98]. Collectively, analysis of the 32 proteins indicates that compared to female neutrophils, male neutrophils have increased phosphorylation of proteins involved in RNA splicing, protein localization, the cytoskeleton, apoptotic signaling in mitochondria, and neutrophil activation.

After incubation with SLIGKV, TMC8 and NUP188 had increased phosphorylation in male neutrophils at 5 minutes, and HNRNPH1 had increased phosphorylation in male neutrophils at 20 minutes. The only protein showing higher phosphorylation in female neutrophils was PRUNE2, and the phosphorylation was only significantly higher at 20 minutes after SLIGKV exposure.

Our observation of sex-based differences in protein phosphorylation suggests that if phosphorylation is considered a general marker for cell activation, then our findings would help explain the observation that male neutrophils respond quicker to the chemorepellent SLIGKV [43]. The slower response time of female neutrophils to SLIGKV could also be due to the elevated levels of the protein phosphatases PPP1R3D and PPP2R2A, and phosphorylated PRUNE2 which suppresses RHOA and AKT signaling, thus reducing cell migration [72, 73, 99, 100]. Our data indicates the surprising finding that many of the sex-based differences in proteins and phosphoproteins are regulators of translation. As these proteins are associated with the translational pathway from spliceosome to ribosomes, it suggests that this is a fundamental process that is underappreciated in neutrophils, especially as it appears to be specific to neutrophils from males. Previous reports also indicate that male neutrophils have significant translation capacity, which may explain why male neutrophils are described as having an “immature” phenotype or possessing “phenotypic plasticity” [35, 100, 101].

The sex-based differences in immune responses, where females have a stronger innate and adaptive immune response to infection but a higher incidence of autoimmune disorders, could in part be explained by our data as male neutrophils respond effectively to a chemorepulsive signal but neutrophils from females do not. In females, this could lead to the persistence of neutrophils at inflammatory sites, which during clearance of bacteria would be beneficial, but in an autoimmune infiltrate the accumulation of neutrophils could lead to a persistent and damaging immune response. An intriguing possibility is that therapies that affect neutrophil biology may need to be modified for male or female patients [13–15, 30–32].

## Conclusion

Human neutrophils have sex-based differences in translation efficiency, protein abundance, and protein phosphorylation. Sex-based differences in translation efficiency did not result in differences in protein levels, suggesting that the differences in translation efficiency may be used to compensate for sex-based differences in the rates at which some proteins are degraded. Sex-based differences in protein levels and protein phosphorylation suggest that male neutrophils might be more ready to adapt to a new environment, and female neutrophils might be more effective at responding to pathogens. In response to the chemorepellent SLIGKV, there was increased phosphorylation of proteins in male neutrophils, but no significantly increased phosphorylation of proteins in female neutrophils. These differences may contribute to the observed sex-based differences in the faster response time of male neutrophils to SLIGKV, and neutrophil-associated disease incidence and severity.

## Data Availability

The datasets used and/or analyzed during the current study are available from the corresponding author on reasonable request. Proteomic data has been uploaded to MassIVE at UCSD Center for Computational Mass Spectrometry with accession numbers MSV000088857 and MSV000094295.

## Highlights

- RNA-seq of monosomes and polysomes indicated that there is more translation of at least 16 mRNAs in human male neutrophils, and more translation of at least 8 mRNAs in female neutrophils.
- 132 proteins were more abundant in male neutrophils and 30 proteins were more abundant in female neutrophils.
- Male neutrophils have more phosphorylation of at least 32 proteins compared to female neutrophils, and we detected no proteins with increased phosphorylation in female neutrophils.
- When neutrophils were stimulated with a chemorepellent for 5 minutes, male but not female neutrophils increased phosphorylation of two proteins.
- Male neutrophils might be more ready to adapt to a new environment, and female neutrophils might be more aggressive.
- These differences may contribute to the observed faster response of male neutrophils to the chemorepellent, and sex-based differences in neutrophil-associated disease incidence and severity.
